# Intensive care unit: results of the Newborn Hearing Screening^☆^^☆☆^

**DOI:** 10.1016/j.bjorl.2015.06.004

**Published:** 2015-10-29

**Authors:** Inaê Costa Rechia, Kátia Pase Liberalesso, Otília Valéria Melchiors Angst, Fernanda Donato Mahl, Michele Vargas Garcia, Eliara Pinto Vieira Biaggio

**Affiliations:** a Human Communication Disorders, Universidade Federal de Santa Maria (UFSM), Santa Maria, RS, Brazil; b Speech Therapy, Universidade Federal de Santa Maria (UFSM), Santa Maria, RS, Brazil; c Sciences Department, Universidade Federal de São Paulo (UNIFESP), São Paulo, SP, Brazil

**Keywords:** Hearing loss, Newborn, Newborn screening, Intensive care units, Perda auditiva, Recém-nascido, Triagem neonatal, Unidades de Terapia Intensiva

## Abstract

**Introduction:**

Procedures for extending the life of newborns are closely related to potential causes of hearing loss, justifying the identification and understanding of risk factors for this deficiency.

**Objective:**

To characterize the population, analyze the frequency of risk factors for hearing loss, and assess the audiological status of infants attended in a Newborn Hearing Screening program (NHS).

**Methods:**

This was a retrospective study that analyzed medical records of 140 patients from a neonatal intensive care unit, identifying the frequency of risk factors for hearing loss and audiological status, utilizing transient otoacoustic emissions and brainstem auditory evoked potential (BAEP).

**Results:**

Prematurity was present in 78.87% of cases; 45% of the infants were underweight and 73% received ototoxic medication. Audiologically, 11.42% failed the NHS, and 5% of cases failed retest; of these, one had results compatible with hearing loss on BAEP.

**Conclusion:**

A higher rate of low birth weight, and prematurity was observed in infants who underwent screening and had an audiological diagnosis by the third month of life. Only one newborn presented a change in audiological status. The authors emphasize the importance of auditory monitoring for all infants, considering this as a high-risk sample for hearing loss.

## Resumo

**Introdução:**

Os procedimentos para prolongamento da vida dos neonatos estão intimamente relacionados com possíveis causas de deficiência auditiva, justificando-se a identificação e o conhecimento dos indicadores de risco para tal deficiência.

**Objetivo:**

Caracterizar a população, analisar a frequência dos indicadores de risco para a deficiência auditiva e verificar o status audiológico de bebês atendidos num programa de Triagem Auditiva Neonatal (TAN).

**Método:**

Estudo do tipo retrospectivo. Foram analisados 140 prontuários da Unidade de Terapia Intensiva Neonatal, caracterizando a população estudada e a frequência dos indicadores de risco para deficiência auditiva e status audiológico, e considerando resultados das emissões otoacústicas transientes e a avaliação diagnóstica por meio do Potencial Evocado Auditivo de Tronco Encefálico (PEATE).

**Resultados:**

Evidenciou-se prematuridade em 78,87% dos casos, 45% exibiam baixo peso e 73% estavam sendo medicados com agentes ototóxicos. Quanto ao status audiológico, 11,42% falharam na TAN. Houve falha no reteste em 5% dos casos e, destes, um neonato apresentou resultado compatível com deficiência auditiva no PEATE.

**Conclusão:**

Houve maior porcentual de prematuros de baixo peso que realizaram a triagem e tiveram um diagnóstico audiológico até o 3° mês de vida. Apenas um neonato apresentou status audiológico alterado. Ressalta-se a importância de acompanhamento auditivo de todos os bebês, considerando esta amostra como de alto risco para deficiência auditiva.

## Introduction

The detection of speech sounds begins in intrauterine life, and is the first step in language acquisition, since it is known that hearing and language are interdependent but interrelated functions. Auditory experiences are of paramount importance, especially before the second year of life, as this is considered the critical period for language acquisition.^1^, ^2^, ^3^ Thus, infants who are born with hearing impairment (HI), are deprived of contact with the world of sound. In this way, the right time for the detection/diagnosis of childhood HI is before the third month of life, and the intervention should begin before the sixth month.^1^, ^4^, ^5^

Studies show that the incidence of significant bilateral congenital HI in healthy neonates is about 1–3 infants/1000 births; conversely, in newborns referred from intensive care units the incidence increases to 2–4%.^5^

There are prenatal, perinatal, and postnatal complications that can cause HI in newborns; these are called risk factors associated with hearing loss (RFHL), namely: the concern of parents with respect to their child general development and the child's hearing, speech, or language development; familial history of permanent deafness; neonatal intensive care unit (NICU) stay >5 days; or occurrence of any associated condition, such as the use of ototoxic medication; congenital infections (rubella, cytomegalovirus, syphilis, herpes, and toxoplasmosis); craniofacial anomalies; genetic syndromes; neurodegenerative disorders; postnatal bacterial or viral infections; head trauma; and chemotherapy.^4^

Hospitalization in NICUs is a very frequent risk factor. Preterm infants are generally underweight, in need for lengthy mechanical ventilation, and may have hyperbilirubinemia at levels that require exchange transfusion, thus making a NICU stay imperative.^6^

The Newborn Hearing Screening (NHS) test is a safe and appropriate procedure for the early detection of HI in neonates and infants.^7^ The current protocol designates as a NHS procedure the recording and analysis of transient-evoked otoacoustic emissions (TEOAE) for neonates without RFHL, and an automatic brainstem auditory evoked potential (aBAEP) study for those who have any RFHL.^4^, ^5^

TEOAE recording is a relatively simple, quick, and objective method for detecting hearing changes of cochlear origin, specifically from the outer hair cells. This method does not quantify the HI, but detects the presence of a cochlear dysfunction.^8^, ^9^, ^10^ BAEP, which is also an objective method, is obtained with surface electrodes that record neural activity generated by the cochlea, auditory nerve, and brainstem in response to auditory stimuli.^5^, ^10^

The NHS outcome criterion is that of “pass and fail”. The “pass” criterion expresses the non-likelihood of HI, and the “fail” criterion expresses the likelihood of HI and the need for a diagnostic evaluation. In case of failure, it is recommended to utilize the BAEP diagnostic procedure for an investigation of electrophysiological thresholds before hospital discharge and/or on the infant's return for retest. If the HI is not confirmed, these infants with RFHL should be followed-up, as they are at an increased risk of difficulties in hearing and in language skill development. If an alteration in BAEP responses is detected, the child will be referred at once for a medical otolaryngological diagnosis and a full audiological evaluation for early rehabilitation.^4^

This study was undertaken because of a need to identify and understand RFHLs; because the increase/advancement of technology associated with the procedures that seek to extend the lives of newborns and infants is closely related to the very factors that can cause hearing impairment.

Thus, this study aimed to characterize the population, analyze the frequency of RFHL, and assess the audiological status of infants treated in an NHS program referred from the NICU of a university hospital.

## Methods

This research is linked to a larger project, called “Child Hearing Impairment: from diagnosis to intervention,” which was approved by the Research Ethics Committee under No. 610,506.

This was a retrospective study,^11^ that aimed to investigate issues related to hearing health in infants referred from the NICU.

The sample analyzed medical records of newborns (NB) and infants who underwent NHS and who came from the NICU during the period from September of 2012 to May of 2013 in a university hospital.

The sample arrangement was made on the basis of eligibility criteria. The inclusion criteria for the analysis of clinical records were as follows: the baby should have been born and remained in the NICU for at least five days, with an NHS carried out in this service. Clinical records with incomplete information and those without an informed consent signed by the parent or guardian were excluded from data collection. This NHS service provides an informed consent explaining to those legally responsible for the child that the data collected on this service may be used for future studies, and that all ethical issues involved will be upheld; nonetheless, the informed consent must be signed at the time of their child care.

Based on a review of medical records, 2097 consultations were retrieved in that period. From this total, 140 medical records were selected, using the inclusion criteria. The following variables were recorded: characteristics of study population (gestational age [GA], birth weight, age at NHS); frequency of RFHLs; audiological status, based on the results documented in the medical record, on an analysis of TEOAE, and on a diagnostic assessment carried out with the use of BAEP; false-positive rate; and prevalence of HI. Such data (presence of RFHL, and TEOAE and BAEP results) were organized into categories of responses, which were stored in a spreadsheet in Microsoft Excel. In the next phase, a data analysis was held with the use of the program PASW Statistic v.18.0 for Windows. In the descriptive analysis, absolute numbers and frequencies of respective variables were sought. For a comparative analysis of the distribution of frequencies, Fisher's exact test and Cochran's test (when three or more categories were present) were used. For all hypothesis tests, a significance level of 0.05 was set. Significant values were marked with an asterisk. All confidence intervals determined during this study were established with 95% of statistical confidence.

## Results

A total of 140 medical records of infants with NICU stay and with mean GA of 34.76 (range: 22–42) weeks were evaluated. Of these, 78.57% (*n* = 110) were preterm newborns with GA <37 weeks; 20.71% (*n* = 29) were term newborns with GA of 37–41 weeks and 6 days; and 0.72% (*n* = 1) was a post-term newborn with GA ≥42 weeks. When the three categories were compared, a predominance of preterm newborns (*p* < 0.001; Cochran's test) was observed.

The mean birth weight of these infants was 2299 g (630–4620). Of these, 39.28% (*n* = 55) had normal weight, that is, ≥2501 g; 45% (*n* = 63) had low-birth weight (1501–2500 g); 12.14% (*n* = 17) had very low birth weight (1001–1500 g); 1.43% (*n* = 2) had extremely low birth weight (751–1000 g); and 2.15% (*n* = 3) were immature infants with <750 g.

The mean age at NHS was 66.06 days (range: 5–492 days). Of these, 77.85% (*n* = 109) underwent screening before the third month of life; and 22.15% (*n* = 31) were tested after the third month of life. Thus, a statistically significant difference between these two groups (*p* < 0.001; Fisher's test) was demonstrated. Due to the tenuous health status of some newborns, NHS was conducted only when their clinical condition became stable. Thus, some children underwent auditory screening in a post-neonatal stage period.

Regarding NHS outcome and characterization of audiological status, 11.42% (*n* = 16) infants failed the first stage of the NHS program carried through TEOAE. Of these 16 babies, 56.25% (*n* = 9) passed the retest, also conducted by TEOAE, and 43.75% (*n* = 7) failed the NHS retest, and were referred for BAEP. Of these, 85.71% (*n* = 6) showed results consistent with normal hearing, and only 14.29% (*n* = 1) showed results compatible with HI. This infant was referred for further medical examination and subsequent speech therapy, including the fitting of hearing aids. Thus, the false-positive rate, that is, the percentage of infants that failed in NHS, but who had normal hearing, was 10.71% (*n* = 15).

The prevalence of pediatric HI in the present study was approximately 0.71:100. Table 1 shows the distribution of frequencies of RFHL in this sample. It is worth noting that that some infants had more than one RFHL.

**Table 1:** Frequency of risk factors for hearing loss (RFHL) associated with hospitalization in a neonatal intensive care unit.

RFHL	Frequency
Family history	5 (3.6%)^a^
Ototoxic medication	103 (73%)^a^
Mechanical ventilation	81 (57.9%)^a^
Hyperbilirubinemia	19 (13.6%)^a^
Congenital infections	3 (2.1%)^a^

a Descriptive analysis.

Table 2 lists the results obtained in the first stage of the NHS program and in NHS retest, according to the RFHL presented.

**Table 2:** Risk factors for hearing loss (RFHL) and audiological status.

	NHS			NHS retest		
RFHL	Passed (*n* = 124)	Failed (*n* = 16)	*p*-Value^a^	Passed (*n* = 9)	Failed (*n* = 7)	*p*-Value^a^
*Ototoxic medication*						
Absent	34 (27.4%)	3(18.7%)		2 (22.2%)	1 (14.3%)	
Present	90 (72.6%)	13 (81.3%)	0.560	7 (77.8%)	6 (85.7%)	1.00

*Mechanical ventilation*						
Absent	52 (41.9%)	7 (43.7%)		5 (55.6%)	2 (28.6)	
Present	72 (58.1%)	9 (56.3%)	1.00	4(44.4%)	5 (71.4%)	0.358

*Hyperbilirubinemia*						
Absent	110 (88.7%)	11 (68.7%)		9 (100%)	2 (28.6%)	
Present	14 (11.3%)	5 (31.3%)	0.044^a^	0 (0%)	5 (71.4%)	0.005^a^

*Congenital infection*						
Absent	121 (97.6%)	16 (100%)		–	–	
Present	3 (2.4%)	0 (0%)	1.00	–	–	

*Family history*						
Absent	119 (96.0%)	16 (100%)		–	–	
Present	5 (4.0%)	0 (0%)	1.00	–	–	

*Drugs/alcohol/smoking*						
Absent	106 (85.5%)	15 (93.7%)		9 (100%)	6 (85.7%)	
Present	18 (14.5%)	1 (6.3%)	0.697	0 (0%)	1 (14.3%)	0.438

NHS, Newborn Hearing Screening.

a Statistically significant values (*p* < 0.05); Fischer's exact test.

## Discussion

Prematurity is not classified as a RFHL by itself, but rather due to the special care in the NICU that these patients generally require, such as the use of ototoxic medication, mechanical ventilation, and specialized consultations.^12^ A recently published study showed that GA and NICU stay at birth are important variables related to the likelihood of hearing screening failure, and that there is a prevalence of HI in preterm newborns. Moreover, in infants with a confirmed HI, the mean GA was 31 weeks.^13^ As for GA, another study also found that the majority of these patients were preterm.^14^ These findings make it possible to infer that, in general, the NICU population consists of underweight preterm newborns in need of specialized care. This study's data agree with these previous studies,^12^, ^13^, ^14^ considering that most of this sample consisted of preterm newborns.

Low birth weight is another factor for alterations in child development among primary neonatal high-risk factors. This study assessed the birth weight of newborns and infants treated at the NICU. The mean birth weight was 2299 g (630–4620) g; 45% (*n* = 63) of the sample had low-birth weight (1501–2500 g). Similarly, another study conducted with a sample of 71 newborns found that, of this total, 52.1% (*n* = 37) had a low weight birth,^15^ which agrees with the present findings.

Regarding the time for diagnosis of HI in NBs, it is a general consensus that such diagnosis should be established early.^5^ It is known that a child with a diagnosis of HI who starts speech therapy before 6 months of life is more likely to develop a proper hearing, and also a better oral language (with statistical significance), when compared to children diagnosed later.^1^ In the present study, the mean age at NHS was 66.06 (5–492) days; 77.15% (*n* = 109) of the sample were submitted to this screening before the third month of life. Babies who failed the first stage of NHS program were referred for retesting, with an audiological diagnosis before completion of the third month of life, which is in accordance with national and international recommendations regarding the time of diagnosis.^1^, ^4^, ^5^

Regarding the outcome of NHS and characterization of audiological status, 11.42% (*n* = 16) of the sample failed the first stage of the NHS program conducted through TEOAE. Of these, 43.75% (*n* = 7) failed a retest. Infants who failed the retest were referred for diagnosis and only 14.29% (*n* = 1) showed results compatible with HI. This finding emphasizes the false-positive rate of the NHS program.

It is recommended that the false-positive rate not exceed 4%.^4^, ^16^ In this university hospital, the false-positive rate was 10.71% (*n* = 15). This high rate can be explained by factors such as excessive background noise, since the room where the NHS is performed does not have sound insulation; and the performance of NHS by an inexperienced staff (as this is a teaching hospital, where students perform their practical activities and are in a process of construction of their clinical practice). Also, the use of TEOAE as the NHS procedure could have had an effect since, although BAEP is the procedure specified for infants with RFHL,^4^ that equipment was not available for carrying out this procedure. The hospital has since purchased this equipment for use by NHS service, thus allowing for NHS in accordance with national and international standards.^4^, ^5^

The findings of the present study are similar to another study that reported a false positive rate of 24.41%,^17^ well above the internationally recommended level.^18^ However, they are in disagreement with the findings of another Brazilian study, that reported a false positive rate of only 1%,^19^ within the target established in the literature. Their rate was possibly achieved by the presence of an otolaryngology service in that hospital's NHS program, by the use of a facilitator auricular maneuver, and by the fact that the procedure was performed by highly specialized speech therapists.

In the present study, the prevalence of HI was 0.71:100 live births. Another Brazilian study found a prevalence of 0.138:100 live births for HI.^19^ Conversely, studies in foreign languages found a prevalence of 2–4% for HI in newborns referred from NICUs.^5^

It is not possible to make inferences regarding this difference, but it is believed that further studies with larger samples would elucidate more precisely the issues related to the occurrence of HI in babies referred from NICUs.

Table 1 shows that the most prevalent of all risk factors was ototoxic medication (73%), which may harm and damage the cochlear function, leading to HI.^20^ The present results were similar to those found in other studies, in which the most frequent risk factor in the populations studied was the use of ototoxic drugs.^21^, ^22^

Due to their health conditions, many newborns need NICU hospitalization and mechanical ventilation. Several aspects have been linked to higher rates of deafness in children submitted to assisted ventilation, including the noise level of the machine, duration of mechanical ventilation, and the lung diseases involved.^23^ In the present study, 57.9% (*n* = 81) of the infants required mechanical ventilation. Of these, 88.9% (*n* = 72) achieved a passing result, and only 11.1% (*n* = 9) failed the initial NHS. These results are consistent with another study of 200 newborns, in which there was a high prevalence of mechanical ventilation. Of these, 84.5% (*n* = 169) achieved a passing result, and 15.5% (*n* = 31) failed the NHS.^23^ Likewise, hyperbilirubinemia has a toxic effect on cochlear hair cells, basal nuclei, and central auditory pathways.^24^ In the present study, 13.6% (*n* = 19) of the sample had hyperbilirubinemia. A study with a sample of 2002 newborns found that 3.9% of this population had some RFHL. Among these, 24.1% had hyperbilirubinemia and 3.9% required mechanical ventilation for over five days.^25^

In Table 2, the authors sought to examine the association between the NHS result in the first stage of the program, and in NHS retest, considering the RFHLs found in this sample. In the first stage of the evaluation, carried out through TEOAE, 81.3% (*n* = 13) of the sample in use of ototoxic medication failed the NHS. This finding is in agreement with another study, in which 8.6% of the infants who failed NHS had some RFHL, and of these, the most prevalent factor was the use of ototoxic medication (42.9%).^25^

Studies show that babies who received exchange transfusion due to high rates of bilirubin may fail the NHS.^25^, ^26^ The structures of the auditory system show high sensitivity to the toxic effects of bilirubin. Among the hearing problems caused by the effects of hyperbilirubinemia, auditory neuropathy, which may (or not) be associated with other hearing disorders, is noteworthy.^27^ In contrast, in the present study, children who presented such RFHL had normal hearing, in accordance with other studies that observed no changes in auditory pathways in newborns with hyperbilirubinemia.^28^, ^29^

Considering this study sample, it was found that among the RFHLs associated with NICU, congenital infection had the lowest occurrence (2.14%). All subjects with this RFHL achieved a passing result in NHS. However, studies show that, even when asymptomatic, congenital infections (rubella, syphilis, cytomegalovirus, herpes, toxoplasmosis, and acquired immunodeficiency syndrome) can cause HI in the neonate and may be associated with late-onset HI and/or with progression of some HI already present at birth.^30^, ^31^

## Conclusion

Of the 140 patients screened during the study period, there was higher incidence of preterm newborns with low birth weight and of NHS and audiological diagnosis performed before the third month of life. The most frequent risk factor was use of ototoxic medication, which is often associated with mechanical ventilation; both were present in the single newborn who presented an audiological assessment consistent with HI. However, in spite of an association between hyperbilirubinemia and “failed” results in tests and retests, BAEP results consistent with normal hearing were noted for all subjects with such RFHL.

Taking into account that this study sample was considered to be at high risk for HI, the authors must emphasize the importance of auditory monitoring until the third year of life of the child, during which hearing changes of progressive- or late-onset character may be identified.

## Conflicts of interest

The authors declare no conflicts of interest.
